# The development of loop-mediated isothermal amplification targeting alpha-tubulin DNA for the rapid detection of *Plasmodium vivax*

**DOI:** 10.1186/1475-2875-13-248

**Published:** 2014-06-30

**Authors:** Sylvatrie-Danne Dinzouna-Boutamba, Hye-Won Yang, So-Young Joo, Sookwan Jeong, Byoung-Kuk Na, Noboru Inoue, Won-Ki Lee, Hyun-Hee Kong, Dong-Il Chung, Youn-Kyoung Goo, Yeonchul Hong

**Affiliations:** 1 Department of Parasitology and Tropical Medicine, Kyungpook National University School of Medicine, 700-422 Daegu, Republic of Korea; 2 Department of Medicine, ROK Army Headquarters, Choongnam, Republic of Korea; 3 Department of Parasitology and Institute of Health Sciences, Gyeongsang National University School of Medicine, 660-751 Jinju, Republic of Korea; 4 National Research Center for Protozoan Diseases, Obihiro University of Agriculture and Veterinary Medicine, 080-8555 Obihiro, Japan; 5 Department of Preventive Medicine, Kyungpook National University Medical Center, Kyungpook National University School of Medicine, Daegu, Republic of Korea; 6 Department of Parasitology, Dong-A University, College of Medicine, 602–714, Busan, Republic of Korea

**Keywords:** Malaria, *Plasmodium vivax*, Immunochromatographic rapid diagnostic tests, Polymerase chain reaction, Loop-mediated isothermal amplification

## Abstract

**Background:**

Malaria that is caused by *Plasmodium vivax* is the most widely distributed human malaria. Its recent resurgence in many parts of the world, including the Republic of Korea (ROK), emphasizes the importance of improved access to the early and accurate detection of *P. vivax* to reduce disease burden. In this study, a rapid and efficient loop-mediated isothermal amplification (LAMP)-based method was developed and validated using blood samples from malaria-suspected patients.

**Method:**

A LAMP assay targeting the α-tubulin gene for the detection of *P. vivax* was developed with six primers that recognize different regions of the target gene. The diagnostic performance of the α-tubulin LAMP assay was compared to three other tests: microscopic examinations, rapid diagnostic tests (RDTs), and nested polymerase chain reactions (PCRs) using 177 whole blood specimens obtained from ROK military personnel from May to December 2011.

**Results:**

The α-tubulin LAMP assay was highly sensitive with a detection limit of 100 copies of *P. vivax* α-tubulin gene per reaction within 50 min. It specifically amplified the target gene only from *P. vivax*. Validation of the α-tubulin LAMP assay showed that the assay had the highest sensitivity (*P* < 0.001 *versus* microscopy; *P* = 0.0023 *versus* RDT) when nested PCR was used as the gold standard and better agreement (concordance: 94.9%, kappa value: 0.865) with nested PCR than RDT and microscopy. A Receiver Operation Characteristics analysis showed that the diagnostic accuracy of the α-tubulin LAMP assay for vivax malaria was higher (Area Under Curve = 0.908) than RDT and microscopy.

**Conclusion:**

This study showed that the *P. vivax* α-tubulin LAMP assay, which can be used to diagnose early infections of vivax malaria, is an alternative molecular diagnostic tool and a point-of-care test that may help to prevent transmission in endemic areas.

## Background

*Plasmodium vivax*, which is a causative agent of human malaria, is the most widely distributed species, and 2.8 billion people are at risk for transmission in the world [1]. Vivax malaria reemerged in 1993 in the Republic of Korea (ROK or South Korea), and its incidence rapidly increased to 4,142 by 2000. *Plasmodium vivax* malaria is commonly believed to be clinically benign and self-limiting [2,3]. However, accumulating lines of evidence have shown that the impacts of *P. vivax* malaria with respect to economic and social burdens in endemic regions have been underestimated [4-6]. Moreover, the resurgence of vivax malaria in many parts of the world, including the ROK [7,8], emphasizes the importance of improving access to reliable diagnostic methods that facilitate the early and accurate diagnosis of malaria, which is urgently required to facilitate disease management and control [9].

Microscopic examinations of Giemsa-stained thick and thin blood films, which are considered the gold standard for the diagnosis of malaria [10,11], are recommended by the World Health Organization. Although this technique is highly specific, its sensitivity for the detection of *P. vivax* is lower than for *Plasmodium falciparum* due to the low parasitaemia of *P. vivax*[12]. Furthermore, it is time consuming, labour-intensive, and requires technical expertise with respect to the interpretation of Giemsa-stained blood smears. Immunochromatographic rapid diagnostic tests (RDTs) that are based on the detection of histidine-rich protein-2 and/or lactate dehydrogenase [11,13] provide rapid and straightforward field tests for the detection of *P. vivax* but are limited in terms of sensitivity and specificity [14,15]. Accordingly, molecular diagnostic methods, such as polymerase chain reaction (PCR) and nested PCR, have been developed and used to improve *P. vivax* detection [16-21]. Although these assays have been shown to be highly effective for diagnosing malaria, they require laboratory equipment, trained personnel, and have long turnaround times, which limit their usefulness for routine diagnoses in the field [22].

Loop-mediated isothermal amplification (LAMP), which is a relatively straightforward and sensitive technique that is based on rapid DNA amplification under isothermal conditions, was recently developed to remove the need for sophisticated and expensive thermal cyclers [23]. LAMP involves the specific amplification of target DNA by *Bacillus stearothermophilus* (*Bst*) DNA polymerase, allowing strand displacement DNA synthesis with a set of six oligonucleotides that recognize independent regions of the target gene. The use of six oligonucleotides improves the specificity and speed of the amplification and forms a loop-structured amplicon, which produces a typical ladder-pattern of multiple bands [24]. A positive reaction is easily determined by eye as turbidity [25] or fluorescence by the inclusion of fluorescent detection dyes, such as SYBR green or hydroxynaphthol under UV light [26,27]. These features allow LAMP assays to be used to detect many pathogenic organisms, such as viruses, bacteria, fungi, parasites, and vivax malaria parasites [28-31].

The present study was undertaken to develop an α-tubulin targeting LAMP assay for the detection of *P. vivax* and to validate the assay using whole blood from suspected malaria patients. The sensitivity and specificity of the devised *P. vivax* α-tubulin LAMP assay were determined and compared with those of microscopy and RDTs with 18S ribosomal RNA (rRNA)-based nested PCR as gold standard. To validate the accuracies of the α-tubulin targeting LAMP assay, the performances of the tests examined were assessed using receiver operating characteristic (ROC) [32,33].

## Methods

### Samples

This study was conducted at Armed Forces Hospitals that treat soldiers stationed near the DMZ, which separates the ROK from the Democratic People’s Republic of Korea (DPRK or North Korea), in the northern part of the Gyeonggi-do Province, in the northwest region of the ROK (between 37°–38° latitude and 127°–128° longitude). This is a high-risk area for malaria and where only *P. vivax* is transmitted [34]. All enrolled soldiers had no history of travel to malaria-endemic areas and had never received a blood transfusion. Whole blood samples were collected by sequential sampling from 177 male ROK soldiers, who provided written informed consent, among all 189 ROK male soldiers who had been admitted to the Armed Forces Hospitals (from May to December 2011) with febrile illness (temperature ≥ 38°C) and were clinically suspected to have malaria. To detect *P. vivax*, approximately 1 mL of blood was collected by venipuncture into a vacutainer tube (Becton Dickinson, Franklin Lakes, NJ, USA) containing ethylenediaminetetraacetic acid (EDTA). Samples were transported on ice within 4 h to a laboratory (Department of Parasitology and Tropical Medicine, Kyungpook National University School of Medicine, Daegu, ROK) where they were stored at -70°C. As is required by the Declaration of Helsinki, donor confidentiality was maintained throughout, and the study was approved by the Ethics Committee of the Armed Forces Medical Command (AFMC-13-IRB-053, July 2011).

### Microscopic examination and rapid diagnostic tests

These samples were assayed using standard diagnostic procedures, including direct microscopic examination of Giemsa-stained thick and thin blood films and RDT. The 177 blood samples from malaria-suspected patients were first examined with RDT and microscopy at the Armed Forces Hospitals. The results for microscopy and RDT were recorded by two different technicians. Then, each sample was blinded and transported to the laboratory (Department of Parasitology and Tropical Medicine, Kyungpook National University School of Medicine, Daegu) for further microscopic examination. A slide was considered negative if no asexual stages of *Plasmodium* spp. were found during examination of 100 fields. Parasite densities were assessed by counting against 200 leucocytes, and converting to parasites per microliter, assuming a standard leucocyte count of 8,000/μL. The immunochromatographic RDT (SD malaria Ag Pf/Pan, Standard Diagnostic, Inc., Hagal-Dong, Korea) detects the parasite antigen *Plasmodium falciparum* Histidine-rich protein-2 (PfHRP-2) specific to *P. falciparum* in one capture site and pan-*Plasmodium* lactate dehydrogenase (pan-pLDH) for all four *Plasmodium* species, in a separate capture line.

### α-tubulin LAMP assay

**Figure 1:**
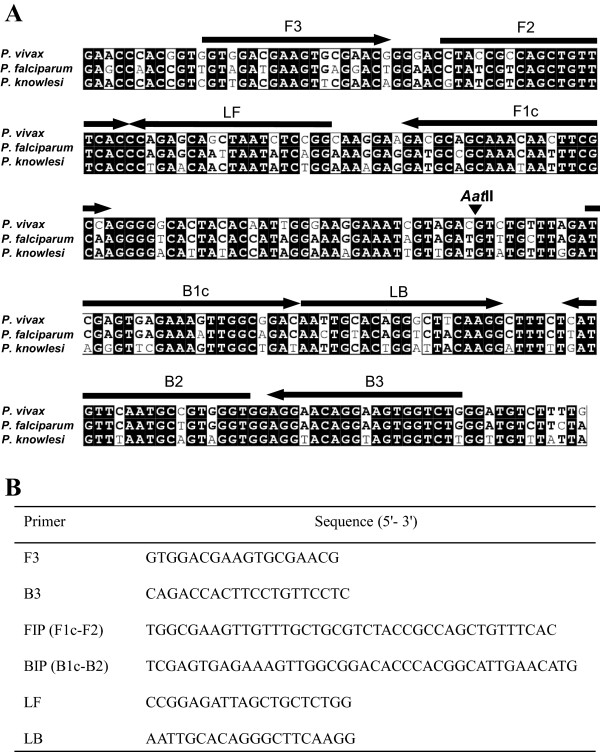
**Primer design for α-tubulin loop-mediated isothermal amplification (LAMP) assay for the detection of *Plasmodium vivax. *(A)** The primers were selected based on a nucleotide sequence alignment of the target region of α-tubulin gene from *P. vivax*, *P. falciparum* [GenBank accession no. XM_001351490] and *Plasmodium knowlesi* [GenBank accession no. XM_002258237] using ClustalW software. Black boxes and bold fonts indicate identical and conserved nucleotides, respectively. The locations of the primer recognition sites are indicated by arrows with the primer names. The black arrowhead indicates the *Aat*II cleavage site. F3, forward outer primer; B3, backward outer primer; FIP, forward inner primer; BIP, backward inner primer; LF, loop forward primer; and LB, loop backward primer. **(B)** Sequences of the α-tubulin LAMP primers.

The *P. vivax*-specific LAMP primers were designed based on previously described using the Primer Explorer program [23,35] (Figure 1). The α-tubulin sequences of *P. vivax* that were used for primer design were retrieved from GenBank [*P. vivax*, GenBank accession no. XM_001615073]. The *P. vivax*-specific α-tubulin LAMP primer set consisted of F3 (forward outer primer), B3 (backward outer primer), FIP (forward inner primer), BIP (backward inner primer), LF (loop forward primer), and LB (loop backward primer). LAMP was performed for 90 min at 64°C in a 25-μL mixture containing 40 pmol each of FIP and BIP, 5 pmol each of F3 and B3, 20 pmol each of LF and LB, 1.4 mM deoxynucleoside triphosphates, 0.8 M betaine, 1 μL of *Bst* DNA polymerase (New England Biolabs, Ipswich, MA, USA) in 2.5 μL of buffer (20 mM Tris–HCl, pH 8.8; 10 mM KCl; 10 mM (NH_4_)_2_SO_4_; 8 mM MgSO_4_; and 0.1% Tween 20), and 1 μL of the plasmid containing the α-tubulin gene fragment or genomic DNA from the whole blood of malaria-suspected patients in a Loopamp real-time turbidimeter (Realoop-30; Eiken Chemical Co., Ltd., Tokyo, Japan). After the LAMP assay, the reactions were inactivated for 2 min at 80°C and then evaluated by electrophoresis in an agarose gel (2.0%) or visualized by a fluorescence detection reagent (FD; Eiken Chemical Co., Ltd.) under UV light. The α-tubulin LAMP using DNA from patients was performed in duplicate. Results from the LAMP assay were independently recorded using the UV lamp in real-time turbidimeter (Eiken Chemical Co., Ltd.) by two investigators who were blinded to the identity of the samples (Table 1 and Table 2). If the results of the duplicate reactions were discrepant, a third amplification and detection were performed to resolve the discrepancy. After scoring the results of fluorescence detection, all the LAMP products were electrophoresed on a 2% agarose gels for detection of LAMP amplification. The results from gel electrophoresis were consistent with those from fluorescence detection.

**Table 1:** Comparison of the results obtained from the four diagnostic methods

**Diagnostic test results**				**No. of cases (%)**
**Microscopy**	**RDT**	**Nested PCR**	**α-tubulin LAMP**	
**+**	**+**	**+**	**+**	96 (54.2)
**–**	**+**	**+**	**+**	23 (13.0)
**–**	**–**	**+**	**+**	5 (2.8)
**–**	**–**	**+**	**+**	4 (2.3)
**–**	**+**	**–**	**+**	8 (3.4)
**–**	**+**	**–**	**–**	10 (5.6)
**–**	**–**	**–**	**+**	1 (0.6)
**–**	**–**	**–**	**–**	30 (16.9)
Total				**177**

**Table 2:** Comparison of the results obtained with the diagnostic methods on the 177 whole blood samples

**Assay**	**No. positive**	**No. negative**	**Sensitivity (95% CI**^**d**^**)**	**Specificity (95% CI)**	**PPV**^**a**^**(95% CI)**	**NPV**^**b**^**(95% CI)**	**% agreement with nested PCR (Kappa)**	**AUC**^**c**^**(95% CI)**
Microscopy	96	81	75.0 (66.8-81.7)	100 (91.1-100)	100 (95.3-100)	60.5 (49.6-70.4)	81.9 (0.624)	0.875 (0.817–0.920)
RDT	137	40	93.0 (87.9–96.4)	63.3 (49.2–75.3)	86.9 (80.1–91.6)	77.5 (62.2–87.8)	84.7 (0.596)	0.781 (0.713–0.840)
Nested PCR	128	49						
α-tubulin LAMP	137	40	100 (96.4-100)	81.6 (68.3-90.2)	93.4 (87.8-96.6)	100 (89.3-100)	94.9 (0.865)	0.908 (0.856–0.946)

The sensitivities and specificities of the tests were determined using the nested PCR results as gold standard.

^*a*^PPV, positive predictive value, ^*b*^NPV, negative predictive value, ^*c*^AUC, area under the receiver operating characteristic curve, ^d^CI, confidence interval.

### Analytical sensitivity and specificity of the α-tubulin LAMP assay

The α-tubulin gene was amplified by PCR from *P. vivax* with the F3 and B3 primers listed in Figure 1. The PCR products were purified with a Qiaquick gel extraction kit (QIAGEN, Inc., Valencia, CA, USA), and the eluted PCR products were cloned into a pGEM-T easy vector (Promega Corporation, Madison, WI, USA). The amplified DNA fragments were completely sequenced (Solgent Co., Ltd. Daejon, Korea) and confirmed to be identical to the sequence of the α-tubulin gene from the *P. vivax* Sal-1 strain. The concentrations of plasmid DNA were measured with a NanoDrop ND-1000 spectrophotometer (NanoDrop Products, Thermo Scientific Instruments, Inc., Wilmington, DE, USA), and the corresponding copy numbers were calculated. The plasmid containing the α-tubulin gene fragment was diluted with TE buffer (10 mM Tris–HCl, 1 mM EDTA) to final concentrations of 1, 10, 10^2^, 10^3^, or 10^4^ copies of the gene per reaction. The specificities of the LAMP assays were evaluated with genomic DNA from *P. falciparum* 3D7, *P. vivax* Sal-1, *Plasmodium malariae* Uganda I/CDC, *Plasmodium ovale curtisi*, *Plasmodium ovale wallikeri, Plasmodium knowlesi* H strain, *Toxoplasma gondii* RH, *Cryptosporidium parvum* Iowa*,* and *Babesia microti* Munich strains. The DNAs of the *Entamoeba histolytica* HM1:IMSS and *Giardia lamblia* WB strains were kindly provided by Dr. Myeong Heon Shin and Dr. Soon-Jung Park (Yonsei University College of Medicine, Seoul, Korea).

### Genomic DNA extraction from whole blood and nested PCR

The DNAs were prepared from 100 μL of whole blood with DNeasy tissue kits (QIAGEN, Inc.). One microlitre of extracted DNA that was dissolved in 20 μL of double-distilled water was used as a template for the nested PCR and LAMP assay. On the other hand, heat-treated lysates of whole blood samples were prepared as previously described with minor modification [36,37]. Briefly, 1 ml of ice-cold 5 mM sodium phosphate buffer (pH 8.0) was added to 20 μl of whole blood sample, vortexed, and centrifuged at 12,000 × *g* for 5 min. The collected pellet was washed two times with 5 mM sodium phosphate buffer (pH 8.0), dissolved with 100 μl of distilled water, and boiled for 10 min. The samples were centrifuged again at 12,000 × g for 2 min, and the supernatants were collected. Four microliter aliquots of the supernatants collected were used as templates for LAMP. The nested PCR based on the 18S rDNA gene was performed as previously described [16] in triplicate with TaKaRa LA Taq polymerase (Takara Bio Inc., Shiga, Japan) in a reaction volume of 20 μL with a thermal cycler (Perkin Elmer Cetus, PerkinElmer Inc., Waltham, MA, USA). Two primer sets for 18S rDNA gene, the universal primer (P1, 5′-ACGATCAGATACCGTCGTAATCTT-3′; P2, 5′-GAACCCAAAGACTTTGATTTCTCAT-3′) and the *P. vivax* specific primer (P1, 5′-ACGATCAGATACCGTCGTAATCTT-3′; V1, 5′-CAATCTAAGAATAAACTCCGA AGAGAAA-3′) that generated a 100-bp product, were employed per reaction. The primer sets that were used had The PCR products from each reaction were confirmed by DNA sequencing (Solgent Co., Ltd.). The amplified products were visualized on 1.5% agarose gels, stained with ethidium bromide, observed with a UV transilluminator, and independently recorded by two experienced investigators blinded against the other methods.

### Statistical analysis

The test sensitivities and specificities were determined using nested PCR as gold standard. The percentage sensitivities and specificities with 95% confidence intervals (95% CIs) were calculated with MedCalc, version 7.0 (MedCalc Software bvba, Ostend, Belgium). Differences in the sensitivities and specificities were compared with McNemar’s normal approximation test with SAS 9.3 (SAS Institute, Inc., Cary, NC, USA). Differences between the areas under the receiver operating characteristic (AUC) were compared with a multiple logistic regression model and SAS 9.3 (SAS Institute, Inc.). The degree of agreement was determined using Kappa statistics in SAS 9.3 (SAS Institute Inc.). Statistical significance was accepted for p values less than 0.001.

## Results

### Optimization of the *P. vivax* α-tubulin LAMP assay conditions

To develop a LAMP assay for the detection of *P. vivax*, a set of six primers targeting the α-tubulin gene was designed (Figure 1). The targeting region of the α-tubulin DNA in this LAMP assay showed relatively low sequence identity among *Plasmodium* spp. (*P. knowlesi* [GenBank accession no. XM_002258237]: 84.0% nucleotide identity; *P. falciparum*, [GenBank accession no. XM_001351490]: 77.6% nucleotide identity)(Figure 1A). The optimal temperature and time for the LAMP reaction were determined with a cloned α-tubulin gene fragment (10^6^ copies per reaction) under isothermal conditions at temperatures of 60°C to 65°C for 120 min by monitoring turbidity. Although amplification targeting of the *P. vivax* α-tubulin gene was detected at all of the temperatures tested, a threshold value of absorbance (0.1) from the LAMP assay was reached most quickly at 64°C (data not shown). No nonspecific amplification was detected in the negative control (plasmid containing no insert) after at least 120 min of incubation. Thus, the subsequent LAMP reactions were conducted at 64°C for 90 min.

### Analytical sensitivity and specificity of the *P. vivax* α-tubulin LAMP assay

**Figure 2:**
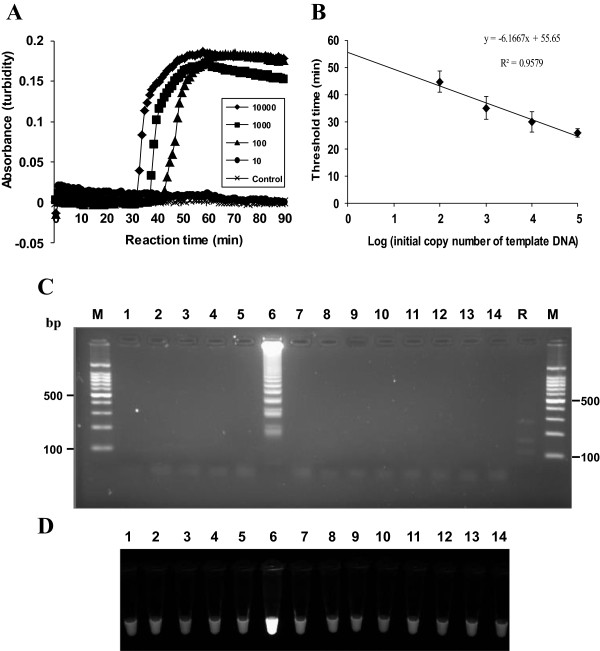
**Sensitivity and specificity of the *P. vivax *α-tubulin LAMP assay. (A)** Sensitivity of the *P. vivax* α-tubulin LAMP assay. Serial 10-fold dilutions of the α-tubulin DNA (10, 10^2^, 10^3^, and 10^4^ copies per reaction) were used for the LAMP assay, and the real-time amplification was monitored by a measurement of absorbance. **(B)** Correlation between the threshold time and the copy number of the α-tubulin DNA in serially diluted samples. The values on the y-axis are the threshold time (in min), which was defined as the time at which the threshold value of absorbance (0.1) was reached. The results show the means and standard deviations of three separate experiments. A plasmid containing no insert was used as a control. The LAMP products were visualized using **(C)** gel electrophoresis and **(D)** Loopamp® fluorescent detection reagent (FD). Lane M, a 100-bp molecular weight marker; lane 1, *P. falciparum*; lane 2, *Plasmodium ovale curtisi*; lane 3, *Plasmodium ovale wallikeri*; lane 4, *Plasmodium malariae*; lane 5, *Plasmodium knowlesi*; lane 6, *P. vivax*; lane 7, *Babesia microti*; lane 8, *Toxoplasma gondii*; lane 9, *Cryptosporidium parvum*; lane 10, *Entamoeba histolytica*; lane 11, *Giardia lamblia*; lane 12, *Trichomonas vaginalis*; lane 13; *Acanthamoeba castellanii*; lane 14, malaria-negative human DNA control; lane R, products of the *AatII* digestion of the LAMP product of α-tubulin.

To assess the sensitivity of the α-tubulin LAMP assay, the LAMP assay was conducted with serially diluted plasmid DNAs containing the α-tubulin gene to the equivalent of 10^5^ to 1 copy per reaction. The LAMP procedure amplified the targeted region at each dilution from the highest copy numbers (10^5^ copies per reaction) (Figure 2B) to as little as 100 copies of *P. vivax* α-tubulin gene per reaction (linear regression coefficient R^2^ = 0.9579), which reached the threshold value of absorbance (0.1) within 50 min (Figure 2A and B). The specificity of the *P. vivax* α-tubulin LAMP assay was evaluated with the genomic DNA of five known malaria-associated species (*P. falciparum, P. malariae, P. ovale curtisi, P. ovale wallikeri, P. knowlesi*) and seven pathogenic but malaria-unrelated species, three belonging to the phylum Apicomplexa (*Babesia microti, Toxoplasma gondii,* and *Cryptosporidium parvum*) and four protozoan parasites (*Entamoeba histolytica, Giardia lamblia, Trichomonas vaginalis,* and *Acanthamoeba castellanii*). As shown in Figure 2C, a typical ladder pattern of the amplified LAMP products with agarose gel electrophoresis was only observed for the *P. vivax* genomic DNA. The *P. vivax* α-tubulin LAMP assay showed no detectable amplification of the other DNAs, including the malaria-negative human DNA controls (Figure 2C). The amplified products of the positive reactions were also visualized with fluorescent detection reagents under UV light (Figure 2D). In order to determine the specificity of the amplification by the α-tubulin LAMP assay, the amplified LAMP products from the *P. vivax* genomic DNA were digested with *Aat*II (Figure 1, arrowhead) and found to generate the expected 187- and 137-bp fragments (Figure 2C, lane R).

### Validation of the *P. vivax* α-tubulin LAMP assay

To validate the α-tubulin LAMP assays, the blood samples of 177 male ROK soldiers who were admitted with febrile illness and who were suspected of having malaria were subjected to α-tubulin LAMP and three available methods, including microscopic examinations, immunochromatographic RDTs and nested PCRs. To determine the accuracy of the tests for diagnosis of *P. vivax* malaria, nested PCR was used as a gold standard and the sensitivity, specificity, positive predictive value (PPV), and negative predictive value (NPV) were calculated for each test. Of the 177 male ROK soldiers, 96 (54%), 137 (77%), 128 (72%), and 137 (77%) were positive for microscopy, RDT, nested PCR, and α-tubulin LAMP, respectively. Microscopic examinations were positive for 96 specimens, resulting in a sensitivity of 75.0% (95% CI, 66.8%–81.7%) and a specificity of 100% (95% CI, 91.1%-100%) (Table 2). The median parasite density in the 96 male patients was 1,263 [103–5,177]/μL. All 96 positive specimens by microscopy were nested PCR positives. Thirty-two of the 81 negative specimens by microscopy were true positives by nested PCR and thus considered false negatives (Table 1). The results obtained by RDT were positive for 137 specimens, resulting in a sensitivity of 93.0% (95% CI, 87.9%–96.4%) and a specificity of 63.3% (95% CI, 49.2%—75.3%) (Table 2). Eighteen of the 137 positive (18/137, 13.1%) by RDT were negative by nested PCR for the detection of *P. vivax* and thus considered false positives. Nine of the 40 specimens (9/40, 22.5%) negative by RDT were true positive by nested PCR and thus considered false negatives (Table 1). All malaria-positive cases by RDT were identified as *P. vivax* infections.

The sensitivity of the α-tubulin LAMP assays was 100% (95% CI, 96.4%–100%), and its specificity was 81.6% (95% CI, 68.3%–90.2%) (Table 2). Eight that were positive by RDT but negative by nested PCR were positive by α-tubulin LAMP (Table 1). Of the 137 positives by α-tubulin LAMP, 128 specimens (136/137, 99.3%) were true positives, and thus 9 specimens were considered a false positive (Table 1). Ten of the 40 negative specimens by α-tubulin LAMP assays were only positive by RDT. On the other hand, α-tubulin LAMP assay conducted using heat-treated lysates produced the same results as those obtained using a commercial kit (DNeasy tissue kit) (data not shown). When nested PCR was used as the gold standard, the α-tubulin LAMP assays had the highest diagnostic sensitivity (*P* < 0.001 *vs.* microscopy; *P* = 0.0023 *vs.* RDT) and better agreement (concordance: 94.9%, kappa value: 0.865) with nested PCR than RDT and microscopy (Table 2). To compare the diagnostic accuracies of microscopy, RDT, and α-tubulin LAMP assays for the detection of *P. vivax*, the diagnostic accuracies were assessed by performing ROC analyses and measuring the AUCs. Among these three diagnostic tests when nested PCR was used as the gold standard, the α-tubulin LAMP assay had the highest AUC (0.908) (*P* = 0.4272 *vs*. microscopy; *P* = 0.0104 *vs*. RDT) among the three tests.

## Discussion

The resurgence of vivax malaria in many parts of the world emphasizes the need for a rapid, sensitive, and inexpensive diagnostic method. Furthermore, it needs to be easily performed and to produce readily interpretable results in the field of an endemic area. As one of the diagnostic methods of vivax malaria, microscopic examinations of blood samples are still widely used in many malaria-endemic areas, including the ROK. As shown in Table 2, microscopic examinations had the lowest sensitivity (75.0%) among the four tests in this study. Furthermore, the requirement of well-trained experts and the long turnaround time limit its usefulness in the field. Due to its convenience, RDT is currently and widely used as an alternative field diagnostic test. However, recent studies suggest that RDT had relatively lower sensitivity and specificity for *P. vivax* than *P. falciparum*[14,15,38]. The RDT in this study had high sensitivity relative to that of the microscopy, and had lower specificity and negative predictive values compared to those of α-tubulin LAMP assays by considering nested PCR as gold standard (Table 2). To solve these problems, molecular diagnostic tests, nested PCRs, and LAMP assays have been developed [11,39]. In particular, *P. vivax* LAMP assays that are based on 18S rDNA, mitochondrial DNA, and Pvr64 have recently been developed and validated for the diagnosis of malaria [28-30,40-44]. The sensitivities of nested PCRs and LAMP assays for *P. falciparum* malaria have been shown to be comparable or higher than RDTs, respectively [45]. Thus, the aim of this study was to design and test the utility of a novel set of *P. vivax*-specific LAMP primers with different target DNA sequences in order to improve diagnostic performance and validate them as an alternative molecular diagnostic test. Although the tubulin gene of *P. vivax* is not species-specific, the β-tubulin gene sequence has previously been used successfully to amplify *P. knowlesi* DNA with a LAMP assay [46]. The targeting region of the α-tubulin DNA in this LAMP assay showed relatively low sequence identity among *Plasmodium* spp. and its use has resulted in no cross reactivity with the other malaria species examined.

In the present study, the diagnostic performance of α-tubulin LAMP assays were compared to three currently available tests, including microscopic examinations, RDT, and nested PCRs using 177 whole blood specimens. When nested PCR was used as gold standard, 128 (72.3%) of the 177 specimens were positive for vivax malaria. PCR is still the most sensitive and specific method for vivax malaria among all of the currently available tests [47]. However, expensive laboratory equipment, the need for a trained specialist, and time-consuming post-PCR procedures, such as agarose gel electrophoresis, limit the usefulness of nested PCR in the field when diagnostic equipment is minimal. However, a LAMP assay does not require a thermal cycler and can be performed with minimal laboratory facilities, such as a heating block or a water bath, and its end points can be determined by turbidity or color development by eye. Furthermore, the straightforward sample preparation for molecular diagnosis reduces the risk of cross-contamination, and thus the risks posed by false-positive results. LAMP assays that are based merely on heat-treated blood have previously been found to be as efficient at detecting malaria parasites as DNA that is extracted with a commercial kit [44,48]. In this study, results of α-tubulin LAMP assay based on heat-treated samples were consistent with those of the LAMP assay using a commercial kit. Thus, the devised *P. vivax* LAMP assay could eliminate the need for DNA extraction without compromising sensitivity and reduce the time required to reach a diagnosis. The α-tubulin LAMP assay had the higher diagnostic accuracy (AUC = 0.908) than microscopy and RDT and showed better agreement with nested PCR (Table 2) and thus can be used in the diagnosis of early infections of vivax malaria as an alternative molecular diagnostic tool.

In endemic countries, including the ROK, vivax malaria is also transmitted by the transfusion of infected blood products [49,50], and, as yet, no reliable approved laboratory test is available for the screening of donated blood. Although the LAMP assay was applied to whole blood samples of patients suspected to be infected, the LAMP assay devised here could also be used for blood screening for *P. vivax*. Furthermore, individuals with low parasite density can provide reservoirs for transmission, and thus an early and accurate diagnostic tool like the α-tubulin LAMP assay may help to prevent transmission in endemic areas.

## Conclusion

This study describes about the development and validation of a LAMP assay for the detection of *P. vivax* DNA in clinical blood samples. In addition, the *P. vivax* α-tubulin LAMP assay developed here can be used to diagnose early infections of vivax malaria, is an alternative molecular diagnostic tool and a point-of-care test that may help to prevent transmission in endemic areas.

## Competing interests

The authors declare that they have no competing interests.

## Authors’ contributions

BKN, NI, HHK, DIC, YKG and YH conceived and designed the study and contributed to the execution of the research. SD and YH wrote the manuscript. WKL contributed statistical analysis. SJ collected the blood samples in the field. SD and HWY carried out the molecular diagnostic tests. HWY, SYJ and SJ performed microscopic examination and RDT. All authors have read and approved the final manuscript.
